# The Next Wave of Immunotherapy: A Critical Review of Nanotechnology Strategies for Immune Checkpoint Blockade

**DOI:** 10.3390/pharmaceutics18040485

**Published:** 2026-04-15

**Authors:** Rashed M. Almuqbil

**Affiliations:** Department of Pharmaceutical Sciences, College of Clinical Pharmacy, King Faisal University, Al-Ahsa 31982, Saudi Arabia; ralmuqbil@kfu.edu.sa; Tel.: +966-555-848-141

**Keywords:** cancers, immunotherapy, immune checkpoint inhibitor, nano-carrier, synergistic effects

## Abstract

The occurrence of cancer is continuing to rise globally and it has been estimated that cancer-related deaths can reach up to 16.3 million by 2040. The immune system has significant contribution in the fight against tumors; nonetheless, the capacity of tumors to escape the strong response induced by the immune system makes cancer a hard-to-treat disease. In recent times, cancer immunotherapy (IMT), particularly immune checkpoint inhibitors (ICPIs), has emerged as an innovative therapeutic approach in the oncology field; however, ICPIs suffer from several drawbacks that limit their therapeutic effectiveness. Therefore, it is crucial to develop a drug delivery system that can increase the therapeutic efficacy of ICPIs and decrease the side effects associated with their use. In order to develop a safe and effective cancer IMT, modifications of nano-biomaterials and development of nanocarrier (NC)-based drug delivery systems can overcome several challenges by improving bioavailability, delivering ICPIs toward targeted areas, enhancing the antitumor immunity, and achieving tumor microenvironment remodeling. NCs also have the potential to exert synergistic effects along with ICPIs. NCs can play a dual role by acting as a targeted delivery system and by combining therapies. The combination of NCs and ICPIs also has the potential to synergize with chemotherapy, radiation, embolization, and tumor ablation to optimize therapeutic outcomes and reduce treatment-associated toxicity. Therefore, this review has focused on revealing the potential of nanotechnology in the field of ICP blockade. In this review, various aspects including NC-mediated targeted delivery of ICPIs and applications of NCs as part of the combination IMT involving ICPIs have been extensively discussed based on numerous potential study findings.

## 1. Introduction

**Figure 1:**
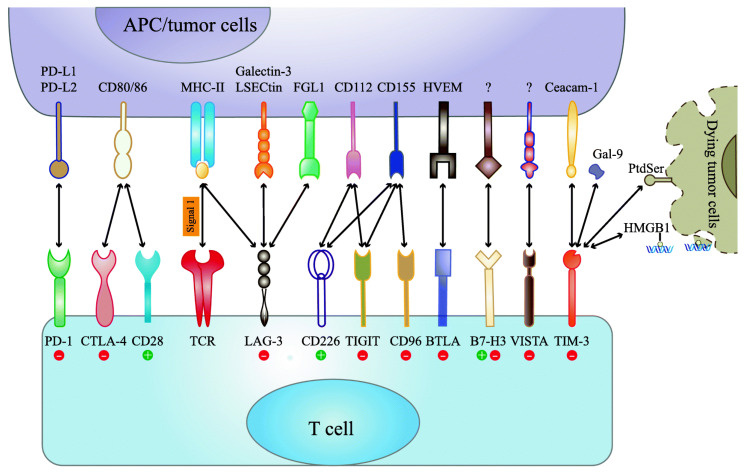
Various immune checkpoint (ICP) receptors and their corresponding ligands. This figure is reproduced from reference [22], Springer Nature, 2019. ICPs are composed of both inhibitory and stimulatory receptors that bind with their natural ligands and can regulate the intensity, type, and duration of immune responses to regulate physiological homeostasis. Nonetheless, cancer cells have the ability to weaken immune responses via over-expressing inhibitory ICP molecules [23]. ICPs, including programmed cell death protein-1 (PD-1) and cytotoxic T-lymphocyte-associated antigen 4 (CTLA-4), bound with their corresponding ligands on antigen-presenting cells (APCs) and/or tumor cells, which can either induce a positive or negative signal to T cell-mediated response [22].

Cancer is a leading cause of death globally, accounting for approximately 10 million deaths in 2020 [1]. This number is estimated to increase up to 16.3 million by 2040 [1,2]. In addition, the occurrence of cancer is continuing to rise, impelled by the growing and aging population as well as alterations in the distribution and occurrence of cancer risk factors. It has been estimated that the number of new cancer cases worldwide will increase in excess of 50% to 30.2 million over the next two decades [2]. The characteristics of cancer cells include evasion of apoptosis, metastasis, insensitivity to antigrowth signals, self-sufficiency of growth signals, limitless replicative potential, and sustained angiogenesis [3]. The immune system plays an important role in the fight against tumors. Nonetheless, the capacity of tumors to escape the strong response induced by the immune system makes cancer a hard-to-treat disease [4]. In recent times, cancer immunotherapy (IMT) has emerged as an innovative therapeutic approach in the oncology field [5]. Immune checkpoint (ICP) is a regulatory protein that modulates immune responses. Once ICPs are presented on cancer cells, ICPs have the capacity to evade immune responses via using their own specific signaling cascades [6]. Immune checkpoint (ICP) molecules expressed on cancer cells can inhibit immune responses against cancer cells [7]. Unlike typical chemotherapies, IMT can trigger a patient’s own immune system via targeting and blocking specific ICP proteins on the surface of cancer cells (Figure 1), which mediates enhanced recognition and attack of cancer cells by the immune system [8]. ICP inhibitors (ICPIs) are a type of IMT that inhibit ICP protein from binding with their corresponding proteins [9].

Although ICPIs have already demonstrated their potential in numerous preclinical and clinical studies, ICPIs are affected by several complications that limit their therapeutic effectiveness. The responses of ICPIs were found to be inconsistent and substantially varied across different types of tumors. Furthermore, the increased occurrences of IMT-associated adverse events including severe inflammation, acquired drug resistance, and organ toxicity also hinder the clinical applications of ICPIs [10]. ICPIs act by releasing brakes of the immune system in the human body, which mediates attack against cancer cells. Nonetheless, this phenomenon can also mediate the attack against normal cells by the immune system, which can further result in various immune-related adverse events that can affect multiple tissues and organs. Significant adverse events associated with ICPIs include neurological disorders (including Guillain–Barre syndrome, myasthenia gravis, and encephalitis), type 1 diabetes, adrenal insufficiency, thyroiditis, hypophysitis, pneumonitis, nephritis, myocarditis, pancreatitis, hepatitis, colitis, vitiligo, pruritus, and skin rash [11].

Therefore, it is crucial to develop a drug delivery system that can increase the therapeutic efficacy of ICPIs and decrease the side effects associated with their use [12]. In order to develop a safe and effective cancer IMT, modifications of nano-biomaterials and development of nanocarrier (NC)-based drug delivery systems can overcome several challenges by delivering ICPIs toward targeted areas, enhancing antitumor immunity, and achieving tumor microenvironment (TME) remodeling [13,14]. Nanotechnology has been widely studied over the past few decades to improve drug delivery. NC-based formulations can effectively regulate the physicochemical features and functions of drugs for targeted delivery and improved therapeutic effects, which can further enhance their on-target therapeutic effects and decrease off-target side effects [15,16,17].

In recent times, NC-based formulations of ICPIs, either entrapped inside NCs or displayed on the surface of NCs, can mediate target receptor binding, tumor accumulation, and antagonistic activities of ICPIs, which has led to enhanced therapeutic effects in preclinical studies [18,19,20,21]. This review has focused on revealing the potential of nanotechnology in ICPI-based therapies. Therefore, in this review, various aspects, including NC-mediated targeted delivery of ICPIs and applications of NCs as part of the combination IMT involving ICPIs, have been extensively discussed based on numerous potential study findings.

## 2. Mechanistic Overview of Immune Checkpoint Blockade

**Figure 2:**
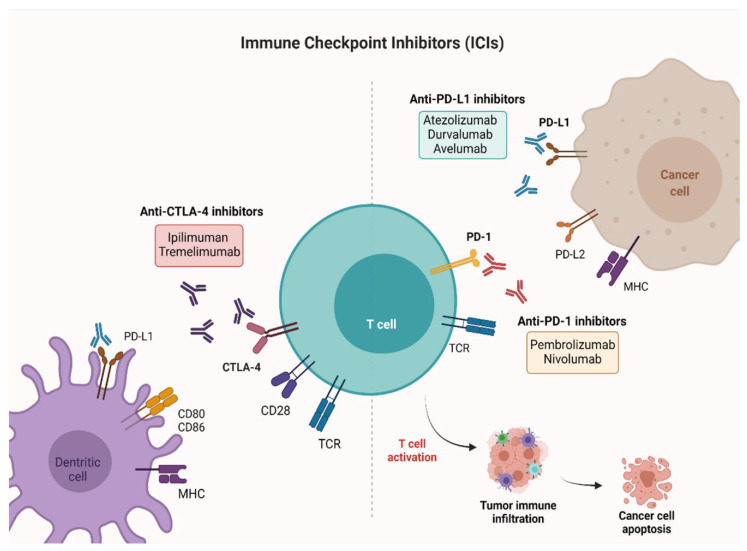
Mechanisms of action of various immune checkpoint inhibitors. This figure is reproduced from reference [37], MDPI, 2021. [Abbreviations: PD-L1, Programmed cell death-ligand 1, CTLA-4, cytotoxic T-lymphocyte-associated antigen 4; PD-1, Programmed cell death-1].

The interactions between the TME and human immune system involve three stages including elimination (which involves destruction of tumor cells by the immune system), equilibrium (where the immune system selects and/or mediates the formation of immunologically resistant tumor cells), and escape (where tumor variants evade the immune system and continue to grow) [24]. It has been revealed that the generation of immunologically cold TME and the subsequent immune escape are reliant on the local immune cells, extracellular matrix, signaling factors, abnormal endothelial cells, and tumor-associated fibroblasts [25,26]. The immunosuppressive TME exhibits an increased level of suppressive molecules, including indoleamine 2,3-dioxygenase (IDO), cluster of differentiation 47, the ICP protein cytotoxic T-lymphocyte protein-4 (CTLA-4), and programmed cell death protein-1 (PD-1)/programmed death ligand-1 (PD-L1) [27,28,29,30,31]. ICPIs induce antitumor immune response by blocking checkpoint proteins from binding with their corresponding proteins [32]. Among the immune-associated responses, activation of T cells and tumor infiltration are most important for the direct eradication of tumor cells [33]. Dendritic cells (DCs) are important APCs that generate the first co-stimulatory signal for priming T cells via detecting and displaying the overexpressed tumor-associated antigens (TAAs) to T cell receptors, which is followed by second antigen-independent signals (suppressive or stimulatory) through ICPs [32]. On the other hand, the second co-inhibitory signal reduces the first activation signal and triggers exhaustion of T cells [34]. It has been observed that tumor cells most commonly display an increased level of suppressive ICP ligands, which can mediate evasion of immune elimination. The interaction between the inhibitory receptor PD-1 on T cells and PD-L1 (the PD-1 ligand) on tumor cells or APCs can markedly suppress activation of T cells and can result in T cell apoptosis, T cell lysis, reduced generation of cytokines, and stimulation of tolerance to antigens, which can help tumor cells to evade immune surveillance [35]. Thus, blocking the interaction between the suppressive receptors and their corresponding ligands can revive effector T cells and therefore induce the capacity of the immune system to fight against tumors [36]. Although there is a growing popularity of small-molecule inhibitors (SMIs), a lack of findings regarding the implications of NCs in modifying their pharmacological activities exists. Figure 2 outlines the mechanisms of action of multiple ICPIs.

## 3. Implications of Nanotechnology in Different Types of Immune Checkpoint Inhibitors

### 3.1. Small-Molecule Inhibitors (SMIs)

SMIs largely dominate the molecular-targeted anticancer therapies, whereas antibody-based biologics are used in IMT. Extensive explorations of monoclonal antibodies (mAbs) against cell surface markers have mediated the comprehension regarding immunoregulatory ligan–receptor pairs [38]. Unlike SMIs, the use of mAbs can be beneficial in the development of targeted therapies against biological targets, which are less likely to cause off-target activities [39]. Nonetheless, mAb-based therapies suffer from several drawbacks. For example, in clinical settings, the mAb infusion regimen is less convenient as compared to orally administrable small-molecule-based therapies. In addition, immune-related adverse events are more manageable in the case of SMIs as compared to mAb-based therapies because of their easier dose adjustment and shorter half-life [38]. These advantages of SMIs over mAb-based therapies have triggered research interests to modulate PD-L1-PD1 pathways [38,40]. Better comprehension regarding the T cell intracellular signaling pathways has reported various negative feedback loops that may be targeted to induce antitumor T cell immunity. Diacylglycerol kinases and mitogen-activated protein kinase 1 are two such noteworthy examples. Interestingly, tyrosine–protein phosphatase non-receptor type 6 (PTPN6), as well as PTPN22 enzymes, and the E3 ubiquitin–protein ligase CBL-B128 were also found to mediate the negative feedback loop [38]. Several SMIs, including GS-4224 and CA-170, are currently being progressed through clinical trials [41,42]. Studies are required to demonstrate the potential use of NCs to deliver such SMIs.

### 3.2. Nucleic Acid-Based Inhibitors

Silencing of ICP genes through small interfering RNA (siRNA) or other suppressive microRNAs can modify the expression of ICP genes and decrease the downstream signaling mechanisms. NCs have the potential to improve the delivery of siRNAs into the tumor-infiltrating lymphocytes and the tumor region. It has been observed that some non-viral vectors including lipid-coated calcium phosphate NCs have the capacity to enhance the entry of siRNA into tumor-infiltrating lymphocytes [43]. Some oligonucleotide-based carriers possess various favorable properties including non-immunogenic, clathrin-based endocytic capacity, stability, lipophilic or amphipathic tendency, cationic charge, and small size. Indeed, polymeric and cationic NCs are likely to possess such features; however, it is challenging to modify and optimize the decoration for targeted and effective delivery. Furthermore, plasmid DNAs can be utilized for localized expressions of PD-L1 trap proteins in association with siRNAs. It is possible to develop PD-L1 traps locally as well as transiently in the TME via plasmid DNA coding the PD-L1 trap through a lipid-protamine-DNA NC, which can also work synergistically with chemotherapies to suppress the growth of tumors [44]. There is an increasing research interest in gene-editing tools that can target the PD-1/PD-L1 axis; however, there are several challenges regarding the safe and effective usage of these tools in clinical studies. An NC-based delivery system was developed by utilizing a low-molecular-weight polyethyleneimine lipid shell and a poly(lactic-co-glycolic) acid (PLGA) core that showed the capacity to encapsulate a PD-L1 gRNA-CRISPR-Cas9 plasmid and transfected human U87 glioma cells expressing PD-L1 [45]. Furthermore, the use of NCs to deliver PD-L1 GFP-CRISPR-Cas9 plasmid into human glioma cells may serve as a potential IMT for the treatment of glioblastoma (GBM) [46].

### 3.3. Antibody-Based Inhibitors

Intraocular signaling pathway and cell–cell contact can induce T cell-mediated immunosuppression because of the generation of transforming growth factor beta and interleukin (IL)-10, as well as overexpression of the transcription factor forkhead box P3, where these molecules serve as important regulators of the immune system [47]. ICPIs have the capacity to neutralize cell–cell contact and trigger apoptosis in the cells that express ICPs. A number of mAb-based therapies, including cemiplimab, pembrolizumab, and nivolumab, has already been approved by the Food and Drug Administration [4]. A range of other anti-PD-1 therapies are currently being studied in clinical studies, including single peptide, immunoglobulin G4, single-chain variable fragment, immunoglobulin G1, and some NC-based formulations. The goal of these innovative anti-PD-1 formulations is to enhance tumor infiltration of these mAbs. It was reported that PLGA-loaded anti-PD-1 NCs can affect CD11c and CD40^+^ populations in mouse tumor models, which can further affect the generation of intratumoral interferon-γ (IFN-γ) and activation of DC_S_, which will eventually result in decreased tumor burden [48]. Table 1 summarizes the potential of various NCs in the delivery of multiple ICPIs.

## 4. Applications of Nanotechnology in Immune Checkpoint Blockade

There is a growing research interest regarding the combination of NCs and ICPIs. NCs provide a wide selection of materials, sizes, and shapes, e.g., polymeric NCs, lipid NCs, metal NCs, and viral or non-infectious plant viral NCs [50,51,52,53]. Because of their tunable and unique physicochemical features, NCs can be effectively used to overcome several limitations of ICI alone. Although various FDA-approved nanotechnologies are available for the delivery of a range of drugs and molecules [54,55,56], various combinations of NCs and ICPIs for cancer treatment are currently being studied in multiple preclinical settings. An NC-based drug delivery system can mediate controlled, protected, and localized release of the ICI cargo. Therefore, it can improve the effectiveness of ICI via enhancing the level of ICI reaching the target area, whilst decreasing toxicity via limiting off-target activities [57]. Furthermore, NCs can co-deliver various cargos to mediate combination therapy [58]. Polymeric thin films can be used to administer NCs, which provide rapid and steady drug release via the buccal route [59,60,61], improving immunological modulation, and being useful in immunotherapeutic systems such as allergen-specific immunotherapy [62]. NCs can also act as immune modulators via enhancing or mimicking immune cell activities [63,64]. This NC-induced immune modulation can be applied in association with ICPIs as part of combinatorial, multifaceted treatment approaches [65]. NCs can be modified to enhance the efficacy of ICPIs or other relevant therapies that can influence their properties, including circulation half-life, solubility, in vivo stability, and buildup in cancer cells or other target areas, e.g., lymphoid tissues for anti-CTLA-4 ICPIs. These properties are affected by the surface chemistry, material composition, shape, surface charge, and size of the NCs [66].

### 4.1. Nanocarrier-Mediated Targeted Delivery of Immune Checkpoint Inhibitors

#### 4.1.1. Nanocarriers for Small Molecule-Based Immune Checkpoint Inhibitors

Small-molecule ICPIs have mainly been developed to overcome the inherent drawbacks of large-molecule-based therapies. The small-molecule ICPIs can be grouped into chemical and peptide-based ICPIs. These ICPIs show improved tissue penetration capacity in comparison with large molecular ICPIs; however, the non-specific distribution of small-molecule ICPIs might lead to systemic toxicity, which can effectively be conquered by using NCs [67]. In a study, Tao et al. [68] identified a PD-1 binding peptide, P-F4, through phage display, which was found to have the capacity to block the PD-1/PD-L1 pathway. The researchers in their study encapsulated P-F4 with mPEG-PLA to enhance the solubility of P-F4 as well as bypass its rapid elimination through enzymes [68]. Chemical ICPIs have the capacity to interfere with the translation or transcription of PD-1/PD-L1, reduce gene expressions, mediate degradations, or competitively bind with the ICP to avert the contact between PD-1 and PD-L1. The NC-based delivery system has the potential for chemical ICPI delivery.

Molecularly imprinted polymers (MIPs) are chemically synthesized alternatives to antibodies that contain tailor-made binding sites that particularly recognize target molecules [69]. MIPs provide unique advantages that can address the shortcomings of antibodies, including high stability, low cost and ease in preparation. MIPs have recently been explored in various applications, including targeted drug delivery, signaling pathway blocking, cell imaging, and disease diagnosis [70,71,72,73,74,75]. In a study, Gu et al. [76] developed a molecularly imprinted polymer-based PD-1 nano inhibitor in order to block the PD-1/PD-L1 pathway. They developed this anti-PD-1 nanoMIP by rationally designing and engineering through epitope imprinting utilizing the N-terminal epitope of PD-1 as the binding site [76]. Most of the chemical- and peptide-based small-molecule ICPIs are currently being studied in various preclinical studies. More studies are also required involving safety and efficacy evaluation as well as experimental validation for their successful translation into clinical settings [67]. In addition, small bioactive molecules like 18β-glycyrrhetinic acid possess anti-inflammatory and immunomodulatory and have shown promise in enhancing the effectiveness of cancer IMT, including ICPIs [77,78,79]. Various studies have demonstrated the promising therapeutic strategy to enhance cancer IMT using the combination of 18β-glycyrrhetinic acid and NCs [80,81,82].

#### 4.1.2. Nanocarriers for Nucleic Acid-Based Immune Checkpoint Inhibitors

There is a growing research interest regarding nucleic acid-based ICPIs, such as aptamers, plasmid DNAs, clustered regularly interspaced palindromic repeats (CRISPR)-Cas, short hairpin RNA (shRNA), and siRNA. NCs can serve as a potential delivery system to overcome several nucleic acid-associated problems, including susceptibility to degradation and inherent instability. Indeed, siRNA is one of the most widely studied nucleic acid-based ICPIs, which inhibits the PD-1/PD-L1 pathway at the post-transcriptional level via decreasing the PD-L1 or PD-1 expression. A range of NCs has already been developed to deliver siRNAs, including lipid- and polymer-based NCs [83,84]. A range of NCs, including dendrimer-entrapped gold NCs, amphiphilic triblock polymers, and noncationic soft polyphenol nanocapsules, have also been developed to encapsulate siRNA against PD-1 (siPD-1) in order to enhance its penetration into tumor tissues and bypass its degradation via mediating the endosomal escape of internalized siPD-1 [85]. Epigallocatechin gallate (EGCG) has the capacity to act as an ICPI of PD-L1 in order to enhance antitumor immune response [86]. In addition, EGCG contains rich aromatic groups and hydroxyl groups to mediate different interactions, such as electrostatic interactions, hydrogen bond interactions, coordination sites, and possible π–π and hydrophobic interactions for biomolecule complexation [87]. In a study, Wu et al. [87] engineered EGCG-based biomimetic nanoassemblies by integrating zinc ions and fluorination into EGCG. The researchers observed that the combination of siRNA for PD-L1 and fluorinated coordinative EGCG mediated antitumor IMT via alleviating the exhaustion of T cells through the modulation of PD-L1 expression in tumor cells [87]. Along with direct siRNA encapsulation, various studies have also evaluated indirect strategies to generate nucleic acids. In this regard, engineered bacteria have been developed to generate RNAs as living factories to effectively generate siRNAs for PD-L1 in cancer cells, which can simplify its manufacturing method and remove complex delivery necessities [88].

The CRISPR-Cas system can effectively knock out PD-1/PD-L1 genes. NCs can provide significant contribution by protecting nucleic acids against degradation to improve their delivery efficiency and stability. A number of factors need to be considered while developing NCs for the transfer of CRISPR-Cas: for instance, the selection of nanoparticles to reduce unspecific in vivo distribution and improve the target-specific secretion and a suitable gene-editing system to enhance the effectiveness of gene editing whilst decreasing off-target activities [89]. An adenovirus was genetically engineered as the vector to deliver CRISPR/Cas9 (sgCas9-AdV) that can encode spCas9 and PD-L1 targeted sgRNA into an injectable silk-gel [90]. The researchers observed that the silk-gel provides protection to sgCas9-AdV against antibody neutralization through the host as well as mediates their prolonged release to enhance gene-editing capacity and therapeutic effectiveness [90]. Furthermore, the silk-gel mediated local sgCas9-AdV retention in tumor tissues and masked them from the host immune system, which further mediated efficient gene transduction over a nine-day period [90]. Zhang et al. [91] loaded a CRISPR/Cas13a system in a dual-locking NC, while the outside of the shell was coated with a layer of a dual-responsive polymer. In tumor tissues, the developed dual-locking NC has the potential to restrict the activation of the CRISPR/Cas13a system by responding to both the acidic extracellular pH and the H_2_O_2_ concentration in the TME [91]. The polymer layer endows the dual-locking NC with a negatively charged and PEGylated surface in normal tissues and during blood circulation, which efficiently preserves its circulation stability and averts the activation of the CRISPR/Cas13a system by suppressing the cellular uptake of the dual-locking NC [91]. Furthermore, the polymer layer was found to be degraded into a cationic polymer after reaching a TME, which mediated cellular internalization of the CRISPR/Cas13a system and activation of gene editing [91]. The developed NC effectively activated T cell-mediated antitumor immunity and TME remodeling in B16F10-bearing mouse models, which eventually led to substantially improved antitumor activities and survival rate [91]. In spite of these promising outcomes, there are several drawbacks/challenges associated with the CRISPR-Cas system, including off-target activities, long-term persistence, immunogenicity, delivery efficiency, and regulatory considerations [92].

Along with the direct downregulation of PD-1/PD-L1 expression, various alternative approaches have also been developed for the regulation of gene transcription, translation, as well as post-translational modification. It is known that NCs have the ability to modify the pharmacokinetic properties of medications and offer passive liver targeting [93]. In a study, Liu et al. [94] reported that nanodelivery of a PD-L1 trap gene showed significant efficacy in the treatment of fibrosis-associated hepatocellular carcinoma in comparison with conventional mAb. They used a lipid-protamine-DNA NC to load plasmid DNA, encoding a trap protein targeting PD-L1 (PD-L1 trap). In addition, PD-L1 trap gene translation produced a trivalent trap protein that showed a markedly higher degree of affinity for mouse PD-L1 as compared to endogenous PD-1; therefore, this trap protein has the potential to play the role of an antagonist to suppress the PD-1/PD-L1 axis [94]. Hydrogels are 3D network structures generated by hydrophilic polymers via physical or chemical cross-linking [95]. Hydrogels are widely used in therapeutic delivery, tissue engineering, and biosensing, owing to their structural similarity to natural biological tissues, excellent biocompatibility, and high water content [95]. In a different study, a DNA polyaptamer hydrogel was developed that can be precisely cut by Cas9/sgRNA for the programmed release of an ICP-blocking DNA aptamer [96]. They also summarized that the developed Cas9/sgRNA-edited ICP-blocking aptamer hydrogel possesses significant capacity to act as an anticancer IMT [96].

#### 4.1.3. Nanocarriers for Antibody-Based Immune Checkpoint Inhibitors

Among the ICPIs, mAbs are the earliest developed ICPIs that are extensively used in clinical settings. However, ICPIs suffer from several drawbacks including suboptimal tumor targeting and poor permeability, which can further lead to on-target off-tumor effects. This phenomenon can seriously damage therapeutic effectiveness and cause immune-related adverse events [67]. Indeed, in order to overcome the abovementioned limitations, NCs can be potentially used to enhance their permeability, tumor targeting capacity, and in vivo distribution [97]. Various nanomaterials, including dendrimers, gold NCs, polymeric micelles, and liposomes, can be used to encapsulate mAbs to reduce their off-target distributions [15]. Moreover, NC-based delivery systems have already been used for anti-PD-1 encapsulation in order to target cancer cells as well as anti-PD-L1 target naive T cells [98]. NCs have also been explored for the delivery of dual-ICPIs. The interaction between B7 molecules and CTLA-4 causes the activation of suppressive signals to suppress excessive activation and proliferation of T cells and circumvent immune system overactivation. Inhibitors of CTLA-4 can induce the activation of T cells by disturbing the interaction between CTLA-4 and its ligands, which can result in improved antitumor immune reactions. On the other hand, simultaneous usage of CTLA-4 and PD-1/PD-L1 inhibitors might significantly induce immune responses via simultaneously enhancing the T cell population and restoring their functions. The goal of NC-mediated delivery of dual inhibitors is to enhance targeting specificity and decrease non-targeted distribution [99,100].

In addition, NCs are utilized for the transfer of dual inhibitors to certain tissues, including the brain. A targeted nanoscale immunoconjugate on natural biopolymer scaffold poly(β-L-malic acid) along with a covalent attachment of a-PD-1 or a-CTLA-4 was developed for systemic delivery through the blood–brain barrier and the activation of local brain antitumor immune responses [101]. In mouse models bearing intracranial GL261 GBM, treatment with the nanoscale immunoconjugates enhanced the level of macrophages, natural killer cells, and CD8^+^ T cells along with a reduction in regulatory T cells (Tregs) in tumor areas of the brain. Furthermore, survival of GBM-bearing mouse models receiving nanoscale immunoconjugates is markedly longer in comparison with the animal models receiving a single checkpoint inhibitor either containing nanoscale immunoconjugates or free a-PD-1 and a-CTLA-4 [101].

### 4.2. Nanocarriers for Combination Immunotherapies with Immune Checkpoint Inhibitors

#### 4.2.1. PD-1/PD-L1 Axis

Anthracyclines are potent inducers of immunogenic cell death (ICD); therefore, the combination of various anthracyclines including epirubicin and PD-1/PD-L1 blockade is currently being studied in clinical studies [102]. Polymeric micelles offer several outstanding advantages, including good stability in biological fluids, water solubility, high loading efficiency, and biocompatibility [103]. In recent times, micellar drug delivery systems are being studied to improve pharmacodynamics and pharmacokinetic profiles and decrease the toxicity of various anticancer therapies [104]. Furthermore, polymeric micelles can enhance the delivery of anticancer drugs to tumors via improving bioavailability and mediating the targeted accumulation of anticancer drugs in solid tumors [105]. In a study, Kinoh et al. [106] revealed that epirubicin-loaded micellar nanomedicines can synergistically act against anti-PD1 antibodies and against phosphatase and tensin homolog (PTEN)-negative and PTEN-positive orthotopic GBM. Furthermore, the combination of anti-PD1 antibodies and epirubicin-loaded micelles surmounted GBM resistance to ICPIs via transforming cold GBM into hot tumors along with increased infiltration of antitumor immune cells by ICD induction, reduction in PD-L1 expression, and abolition of immunosuppressive myeloid-derived suppressor cells on tumor cells [106]. In recent times, various preclinical studies are evaluating the efficacy of the NC-mediated delivery of radiotherapy or chemotherapy in combination with IMT. Researchers developed doxorubicin-loaded high-density lipoprotein-mimicking nanodiscs, which can mediate ICPI in murine tumor models [101]. The researchers observed that doxorubicin delivery through nanodiscs induced ICD of cancer cells and exerted antitumor effects without any apparent off-target activities. Furthermore, the combination of cancer IMT with nanodiscs and anti-PD-1 therapy mediated complete regression of established MC38 and CT26 colon carcinoma tumors in 80–88% of mouse models and provided protection to survivors against cancer recurrence [101].

Nanoscale coordination polymers (NCPs) encompassing both amorphous coordination polymer NCs and crystalline nanoscale metal–organic frameworks have gained research interest as adaptable hybrid materials. NCPs have adjustable composition, surface functionality, and porosity. Indeed, these properties mediate precise control over biological interactions, release timing, and drug loading [107]. In a study, Duan et al. [58] demonstrated effective IMT of colorectal cancer through systemic delivery of an immunostimulatory chemotherapeutic combination in NCP core–shell particles. They loaded the nanoscale particles with dihydroartemisinin and oxaliplatin. These drugs can synergistically generate reactive oxygen species (ROS) and mediate anticancer effects. This synergistic action towards ROS generation mediated immune activation to synergize with an anti-PD-L1 antibody to treat murine colorectal cancer tumors. Furthermore, the favorable tumor uptake and biodistribution of NCPs and the existence of peripheral neuropathy allowed repeated dosing to achieve 100% tumor eradication. Collectively, the involvement of both adaptive and innate immune systems mediated long-lasting and robust antitumor immunity, which further prevented the formation of tumors when cured mice were challenged with cancer cells [58]. The NCPs offer multiple delivery features, which involve flexible composition as well as biodegradability within tissues. He et al. [108] developed immunogenic NCs to induce the antitumor action of anti-PD-L1 antibody-induced cancer IMT. Their developed NCPs were composed of photosensitizer pyropheophorbide–lipid conjugate (pyrolipid) in the shell (NCP@pyrolipid) and oxaliplatin in the core for effective photodynamic therapy (PDT) and chemotherapy. The combination of pyrolipid-induced PDT and oxaliplatin synergistically induced immune responses and destroyed tumor cells further resulted in the exposure of calreticulin on the cell surface, antitumor vaccination and an abscopal effect. In combination with anti-PD-L1 therapy, the developed NCP@pyrolipid-induced regression of non-irradiated distant tumors and light-irradiated primary tumors via mediating a strong tumor-specific immune response.

A major challenge with NC-based immunomodulatory vaccine platforms is the tendency of phagocytes to sequester NCs, which can result in accumulation in the spleen as well as liver and poor delivery in target tissues [109]. Luo et al. [110] developed a nanovaccine containing a mixture of an antigen and PC7A NC (a synthetic polymeric NC), which showed robust cytotoxic T cell responses along with low systemic expressions of cytokines. PC7A NC effectively mediated cytosolic delivery of tumor antigens to antigen-presenting cells in draining lymph nodes to raise the level of surface presentation while concurrently activating type I interferon-stimulated genes. This action was found to be reliant on the STING pathway instead of the mitochondrial anti-viral-signaling protein or Toll-like receptor (TLR) pathway. The developed nanovaccine generated strong suppression of tumor growth human papilloma virus-E6/E7, colon cancer, and melanoma tumor models. The combination of anti-PD-1 therapy and PC7A nanovaccine exhibited outstanding synergistic effects, which mediated 100% survival over 60 days in a TC-1 tumor model. In addition to this, complete inhibition of tumor growth was observed following rechallenging these tumor-free animals with TC-1 cells, which indicates the generation of long-term antitumor memory. The researchers concluded that the developed STING-activating nanovaccine can be a potential, robust, and safe approach in inducing antitumor immunity for cancer IMT [110].

There is a growing popularity of gold nanostars as promising platforms for therapeutic applications, bioimaging, and biosensing, because of their biocompatibility, strong plasmonic enhancement and tunable optical features. Liu et al. [111] developed an innovative two-pronged modality known as synergistic immuno photothermal nanotherapy (SYMPHONY), which can safely and effectively eradicate distant metastatic foci and primary tumors. The combination of ICPI and plasmonic gold nanostar-mediated PDT was found to be effective in completely eradicating both distant untreated tumors and primary-treated tumors in some mouse models that were implanted with the MB49 bladder cancer cells. Moreover, SYMPHONY cured the delayed rechallenge with MB49 cancer cells in mouse models, which did not result in the formation of new tumors following 60 days observation, suggesting the potential of SYMPHONY in inducing long-lasting protection against MB49 cancer cells. In a different study, Huang et al. [112] developed a liposome-based photothermal therapy (PTT) NC through the self-assembly of intravenously injectable lipids and indocyanine green (ICG), a photothermal agent. The developed ICG-liposome exhibited enhanced ICG encapsulation efficiency (over 95%), long-term storage stability, and improved near-infrared (NIR) light-triggered photothermal reaction in vitro as well as in vivo. In addition, ICG-liposome markedly destroyed the primary tumor following laser irradiation in MC38 and CT26 mouse colon cancer models, and mediated CD8 T cell infiltration in distant tumors. The researcher also combined T cell immunoglobulin and mucin domain containing protein 3 (TIM-3) and PD-1 blockade along with PTT in an MC38 mouse colon cancer model. The combined therapy effectively eradicated the primary tumors, which generated systemic immune responses and suppressed the growth of distant tumors. Collectively, the combination of TIM-3/PD-1 blockade and ICG-liposome has great future potential in cancer IMT [112]. Ou et al. [113] developed a plug-and-play nanorization, ultrasonic bubble bursting of coarse black phosphorus flakes for the continuous generation of black phosphorus nanosheets. The developed nanosheets were used to load chitosan–polyethylene glycol (a targeting agent) doxorubicin and cancer growth inhibitor (PD-L1 siRNA) to achieve effective chemo-photoimmunotherapy against colorectal cancer. The nanosheet surfaces were first loaded with doxorubicin, folic acid (for targetability), and chitosan–polyethylene glycol (for electrostatic loading effect) to generate a chemo-phototherapeutic sub-platform, which was further encapsulated with PD-L1 siRNA to develop a nanoformulation termed as BP-DcF@sPL. The developed BP-DcF@sPL induced chemo-phototherapeutic effects, including local ROS and heat generation and necrosis/apoptosis, which interfered with PD-L1 pathway-regulated immune tolerance and inhibition of CD8^+^ T cells. Furthermore, the NIR-mediated DC maturation ameliorated lysis effects because of the induction of T cell infiltrations into the TME. Treatment with this nanoformulation also prolonged the survival period of the treated C57BL/6 and BALB/c nude mouse models [113].

#### 4.2.2. CTLA-4 Pathway

Antigen availability and presentation are important for the activation of cancer immune response to trigger tumor ablation with consequent tumor antigen release and to mediate the activation of the innate immune system. PLGA-based drug delivery systems offer a range of advantages. PLGA is completely biodegradable, which removes the need to remove empty remnants following complete drug release [114]. Furthermore, PLGA breaks down into its non-toxic monomers, including glycolic and lactic acid, which mediates excellent biocompatibility of the systems. PLGA-based drug delivery systems can control drug release kinetics for a flexible period of time, which can range from a few hours to several months [115,116]. In a study, Chen et al. [117] developed a combination of NC-based photothermal therapy with ICPI. The NC-based formulations were composed of PLGA (for encapsulation), ICG (to mediate photothermal therapy), and imiquimod (R837) (as TLR7 agonist [117]. The generated PLGA-ICG-R837 NCs were made solely of three clinically approved components that can be utilized for NIR laser-triggered photothermal ablation of primary tumors, producing TAAs, which in the presence of R837-containing NCs as the adjuvant can exert vaccine-like activities. Moreover, robust immune–memory response was seen 40 days after NC-mediated photothermal tumor ablation, which along with anti-CLTA4 therapy might provide significant protection to mice against tumor rechallenge. The researchers also concluded that in association with the CTLA-4 checkpoint blockade, the mediated immunological responses are likely to attack remaining tumor cells in mouse models, which is beneficial in metastasis suppression, and may potentially be applicable for various types of tumor models [117].

NC has the potential to improve the release and presentation of tumor antigens in the case of both PTT and PDT, which further enhances the efficacy of ICPIs. In a different study, Xu et al. [118] developed upconversion NCs (UCNCs) that have the capacity to simultaneously load R837 and chlorin e6 (Ce6). It was reported that UCNC-Ce6-R837 NCs under NIR irradiation with improved depth of tissue penetration might provide PDT-mediated tumor destruction to produce a pool of TAAs, which in the presence of R837-loaded NCs as the adjuvant are capable enough to mediate robust immune responses against tumors. In addition, the combination of PDT along with the UCNC-Ce6-R837 and CTLA-4 ICP blockade exhibited excellent effectiveness in eradicating tumors exposed to the NIR laser and also mediated robust immune responses against tumor to suppress the growth of left-over distant tumors following the PDT therapy. This unique IMT approach can provide a long-term immune memory function to provide protection to treated mice against tumor cell rechallenge. Collectively, this study reveals an immune-inducing UCNC-based PDT approach in association with CTLA-4 ICP blockade to efficiently eradicate primary tumors under light exposure, which can further result in the suppression of distant tumors that are hard to reach by light, and the prevention of tumor recurrence through the immune memory response [118].

Immunoglobulin G-opsonized PLGA NCs are promising biodegradable carriers for targeted administration of protein antigens, allowing efficient cross-priming in vivo [119]. Chen et al. [120] developed core–shell PLGA NCs by encapsulating catalase (Cat), an enzyme that can generate O_2_ by decomposing H_2_O_2_ in the core, as well as loaded R837 within the PLGA shell. The developed NCs were found to significantly improve the efficacy of radiotherapy via regulating the immune-suppressive TME and relieving the tumor hypoxia. It was observed that TAAs resulted in postradiotherapy-mediated ICD in the presence of such R837-loaded NCs may mediate robust immune responses against tumors, which along with CTLA-4 ICP blockade is likely to efficiently suppress tumor metastases through a strong abscopal effect. In addition to this, after this treatment, a long-term immunological memory response provided protection to mouse models against tumor rechallenge. Collectively, the study findings indicate the potential of this novel NC-based therapeutic strategy to mediate synergistic systemic responses following a local radiotherapy.

**Figure 3:**
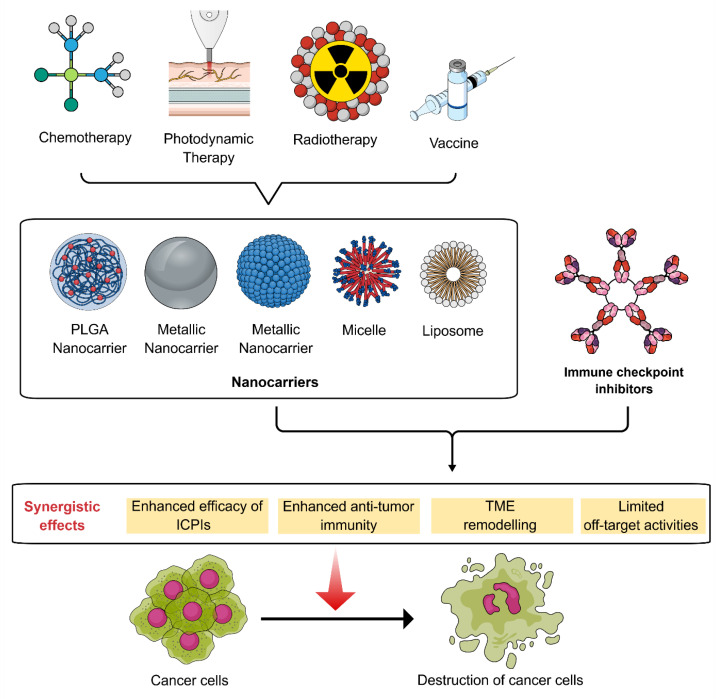
Synergistic effects exerted by the combinations of nanocarrier-based drug delivery systems and immune checkpoint inhibitors. [Abbreviations: PLGA, poly(lactic-co-glycolic) acid; ICPIs, immune checkpoint inhibitors; TME, tumor microenvironment].

In a different study, Chen et al. [121] revealed that iron-oxide NCs (IONCs) have the potential to be used as biodegradable nanomediators for PTT. Polymer-coated IONCs were generated via thermal decomposition of iron oxide (III) in the presence of 1-octadecene and oleic acid. The researchers observed that PTT along with IONCs can synergistically cause depletion of immunosuppressive Tregs in 4T1 murine breast tumors in order to overcome TME-caused immunosuppression and can markedly induce the anticancer effect of ICPI-based cancer IMT. Flow cytometry was used to assess the population of Tregs and CD8^+^ T cells. Tumor growth following combination therapy of both PTT and anti-CTLA-4 was further evaluated. It was also observed that IONC-mediated PTT can result in the eradication of tumor-associated Tregs. When combined with ICPI therapy, IONC-mediated sequential PTT markedly suppressed 4T1 tumor growth and produced an abscopal effect on suppressing distal tumor growth. Moreover, the combination therapy involving IONC-mediated sequential PTT has the potential to induce memory tumor antigen-specific CD8^+^ T cells to avert the recurrence of tumor cells [121]. Figure 3 illustrates the synergistic effects exerted by the combinations of NC-based drug delivery systems and ICPIs.

## 5. Preclinical and Clinical Studies Regarding the Combination of Nanotechnology and Immune Checkpoint Inhibitors

Growing number of findings obtained from preclinical studies (Table 2) and clinical trials (Table 3) are mediating the translation of NC-based formulations from laboratory to clinical settings [122]. Preclinical studies involving xenograft and syngeneic tumor models have revealed that nano-immunoconjugates have the capacity to markedly improve the efficiency of ICPIs via reprogramming the immunosuppressive TME, decreasing off-target effects, and enhancing tumor accumulation [123]. In this regard, for instance, PLGA NCs co-loaded with CpG oligonucleotides and anti-PD-1 antibodies showed significant tumor regression in murine melanoma models in comparison with systemic administration, along with enhanced levels of IFN-γ secretion and CD8^+^ T cell infiltrations [124]. On the other hand, mesoporous silica NCs co-loaded with PD-L1 siRNA and IDO inhibitors exerted synergistic antitumor responses via ICP silencing and TME modulation [125]. Interestingly, imaging-integrated platforms including ^64^Cu-labeled PEGylated immunoliposomes also showed specific PET-based quantification of biodistribution, which mediated favorable pharmacokinetic properties for dose optimization [126]. A clinical trial is also currently ongoing (NCT03961698) involving ICPIs, which is studying the effects of albumin-bound anti-PD-L1 nanoconjugates in metastatic breast cancer patients. Tumor-targeted NCs containing imaging probes and anti-CTLA-4 therapy are also being studied for intratumoral treatment of GBM [127]. Even though there is still no approved ICPIs available that are formulated as nano-immunoconjugates, their enhanced therapeutic outcomes, improved immunogenic modulation, and favorable preclinical safety profiles strongly indicate their potential in clinical translation [122]. However, various challenges are currently hindering their successful translation, including regulatory ambiguity surrounding multifunctional theranostics, large-scale reproducibility, and immunogenicity of NC materials. Indeed, it is crucial to overcome these issues with biomarker-guided trial design, good manufacturing practice-compliant synthesis, and standardized protocols for further development of nano-immunoconjugates into next-generation therapy in the field of clinical immuno-oncology [122].

## 6. Current Challenges and Future Perspectives

IMT is a novel therapeutic approach in cancer treatment, which activates the immune system and improves host immune responses to inhibit the growth as well as metastasis of cancer cells. Indeed, NCs can deliver IMTs to cancer cells and enhance their anticancer immune responses owing to the unique properties of NCs including controlled release, excellent biocompatibility, and targeting [129]. NCs can effectively decrease the ICPI-associated side effects. Furthermore, NCs can be used to deliver ICPIs in order to improve the bioavailability of these drugs. Nanomaterials like hydrogels, through precise delivery, can decrease systemic exposure of ICPIs, which can further decrease the occurrence of immune-associated adverse events [130]. NCs can also ameliorate the persistence of ICPIs. For example, an injectable nanohydrogel has been developed by researchers via combining carboxymethyl chitosan and bioadhesive NCs based on polylactic acid-hyperbranched polyglycerol, which can maintain the effects of ICPIs through continuous release of drugs over one week [131]. Various physicochemical features of NCs, including surface charge, shape, and size, have profound effects on their stability and mode of binding with ICPIs, having an impact on the drug release pattern of ICPIs and their therapeutic efficacy. For example, complexes formed by NCs with overly small sizes following conjugation with antibodies might suffer from reduced stability. Since NCs often tend to have relatively high surface energy, thus NCs are susceptible to aggregation or dissociation within the physiological condition. Furthermore, such instability can result in the detachment of antibodies from the surfaces of NCs [132]. NCs of extremely small sizes might get rapidly removed from the body via renal filtration, which results in reduced circulation time in vivo and thus diminished efficacy of drug delivery [133].

There are two major long-term safety issues of NCs including the synergistic toxicity caused by their combined use and the potential toxicity of NCs themselves. Some NCs might accumulate in the body, which can result in tissue damage and chronic inflammation. For instance, synthetic amorphous silica nanoparticles can accumulate in cells after entering the body via passive diffusion and active endocytosis, which can further result in the distribution in nearly all organs, and to direct or indirect tissue damage and inflammation [134]. Moreover, ICPIs themselves might result in immune-associated adverse events, including endocrine disorders and autoimmune diseases. A combination of NCs might further exacerbate these adverse events or induce new toxic effects. For instance, NCs might alter the metabolism and distribution of ICPIs in the body, which can lead to extremely high concentrations of the drug in some tissues and increase the risk of adverse events. In addition, the interaction between ICPIs and NCs might also influence the balance of the immune system, which can cause extreme immunosuppression or immune activation [135].

NCs might also be detected as foreign substances by the immune system, which can mediate an immune response that can lead to their rapid clearance. The induced immune response might further trigger tissue damage and inflammation, which can compromise a patient’s health. In this regard, for instance, the amino groups on the surface of polyethyleneimine NCs provide them with a strong positive charge. They rapidly and readily bind with serum proteins once they enter into the body, which can mediate strong complement activation and a strong immune response. On the other hand, lipid NCs can adsorb plasma proteins in the bloodstream, which can result in the formation of a protein corona. Interestingly, this formation modifies the particle size and surface charge of NCs, which can further affect their targeting capacity and distribution [136]. Therefore, future studies should focus on more rigorous innovation and research in the development of ICPIs and NCs, and the design as well as development of novel NCs. Natural NCs, including peptide assemblies and cell membrane-derived vesicles, can also be considered and improved to improve their safety and therapeutic efficacy [137]. More studies are required to develop NCs containing lower toxicity and enhanced targeting specificity, and to design and develop novel ICPIs that are able to overcome tumor immune escape. Future studies are also required to explore novel therapeutic targets and mechanisms of action, which are likely to prove novel and improved methods and perspectives for the development of cancer IMT.

## 7. Conclusions

ICPIs have revolutionized the cancer IMT and clinical management of various types of cancers; however, there are significant prospects to minimize their toxic effects, decrease recurrence, and enhance therapeutic outcome. There is a substantial potential to overcome the limitations of ICPIs by combining ICPIs with NCs. NCs have the capacity to play a role as a targeted delivery system for ICPIs alone or as part of a combination with various other therapies. Furthermore, NCs also have the potential to exert synergistic effects along with ICPIs. NCs can play a dual role by acting as the targeted delivery system and by combining therapies. The combination of NCs and ICPIs also has the potential to synergize with chemotherapy, radiation, embolization, and tumor ablation to optimize therapeutic outcomes and reduce treatment-associated toxicity. Although a number of preclinical studies have already demonstrated their potential, more clinical studies are required for the successful translation and use of combination of NCs and ICPIs in clinical settings.

## Figures and Tables

**Table 1:** A summary of preclinical studies that has indicated the potential use of nanocarriers in delivering various immune checkpoint inhibitors.

Nanocarrier (NC) Type	Immune Checkpoint Inhibitor (ICPI) Type	Study Model	Study Outcomes	References
Cationic core–shell nanocarrier (NC)	CRISPR/Cas9 pDNA to knockdown anti-programmed cell death protein-1 (PD-1)	In vitro and in vivo	NCs were rapidly taken up by U87 cells and entered into the nucleus; resulted in the expression of PD-L1 gRNA and Cas9 enzyme	[46]
Lipid-protamine-DNA NC containing programmed death ligand-1 (PD-L1) trap	Anti-PD-L1 monoclonal antibody	Mouse orthotopic 4T1 breast tumor model; B16-F10 mouse melanoma model	As compared to the combination of anti-PD-L1 mAb and oxaliplatin, the combination of PD-L1 trap and oxaliplatin did not induce accumulation of Th17 cells in the spleen, which suggests improved tolerance and lower chances of immune-related adverse effects	[44]
Poly(lactic-co-glycolic) acid (PLGA) NCs	Anti–PD-1 antibody	B16-F10 mouse melanoma model	Attenuation of the dosage of NCs prevented toxicity and markedly enhanced its antitumor action in mice; DCs internalized NCs in the spleen, which resulted in their maturation and the subsequent T cell activations	[49]

**Table 2:** A summary of preclinical studies that evaluated synergistic effects mediated by various combinations of nanocarriers and immune checkpoint inhibitors.

Nanocarrier (NC) Type	Immune Checkpoint Inhibitor (ICPI) Type	Cancer Model	Therapeutic Outcomes	References
Poly(lactic-co-glycolic) acid (PLGA)-indocyanine green-imiquimod (R837) NCs	Anti-cytotoxic T-lymphocyte antigen-4 (CTLA-4)	Mouse 4T1 breast tumor model, CT26 mouse colon carcinoma model	Robust immune–memory response, protection to mice against tumor rechallenge.	[117]
PLGA-R837@catalase (Cat) NCs	Anti-CTLA-4	CT26 mouse colon carcinoma model	Significantly improved the efficacy of radiotherapy via regulating the immune-suppressive TME and relieving the tumor hypoxia.	[120]
Iron-oxide NCs (IONCs)	Anti-CTLA-4	Mouse 4T1 breast tumor model	Photothermal therapy along with IONCs synergistically depleted immunosuppressive regulatory T cells in the mouse model to overcome TME-caused immunosuppression, markedly induced anticancer effects of ICPI, significantly suppressed 4T1 tumor growth, and produced an abscopal effect on suppressing distal tumor growth.	[121]
Upconversion NC-chlorin e6 (Ce6)-R837	Anti-CTLA-4	CT26 mouse colon carcinoma model	Showed significant efficacy in eradicating tumors; mediating robust immune responses against tumors to suppress the growth of left-over distant tumors, provided protection to treated mice against tumor cell rechallenge.	[118]
Doxorubicin-loaded nanodiscs	Anti-PD-1	MC38 and CT26 colon carcinoma in mouse model	Combination of nanodiscs and anti-PD-1 therapy mediated the complete regression of established MC38 and CT26 colon carcinoma tumors in 80–88% of mouse models, which provided protection to survivors against cancer recurrence.	[101]
Epirubicin-loaded micelles	Anti-programmed death ligand-1 (PD-L1)	Mouse model of glioblastoma (GBM)	Surmounted GBM resistance to ICPIs via transforming cold GBM into hot tumors along with increased infiltration of antitumor immune cells by immunogenic cell death induction, reduction in PD-L1 expression, and abolition of immunosuppressive myeloid-derived suppressor cells on tumor cells.	[106]
Nanoscale coordination polymer (NCP) core–shell particles loaded with nanoscale particles with dihydroartemisinin and oxaliplatin	Anti-PD-1	MC38 and CT26 colon carcinoma in mouse model	Synergized with an anti-PD-L1 antibody to treat murine colorectal cancer tumors, mediated long-lasting and robust antitumor immunity, complete tumor eradication.	[58]
NCPs composed of photosensitizer pyropheophorbide–lipid conjugate (pyrolipid) in the shell (NCP@pyrolipid) and oxaliplatin in the core	Anti-programmed death ligand-1 (PD-L1)	CT26 colon carcinoma in mouse model, human colorectal adenocarcinoma HT29 cells	Synergistically induced immune responses and destroyed tumor cells, induced regression of non-irradiated distant tumors and light-irradiated primary tumors.	[108]
Indocyanine green (ICG)-liposome	Anti-PD-1, anti-TIM-3	MC38 and CT26 mouse colon cancer models	Markedly destroyed the primary tumor following laser irradiation in animal models, mediated CD8 T cell infiltration in distant tumors.	[112]
PC7A NC	Anti-PD-L1	B16-OVA mouse model for melanoma, MC38 colon carcinoma in mouse model	Showed robust cytotoxic T cell responses along with low systemic expressions of cytokines. A combination of anti-PD-1 therapy and PC7A nanovaccine mediated 100% survival over 60 days in a TC-1 tumor model, complete inhibition of tumor growth was observed following rechallenging these tumor-free animals with TC-1 cells.	[110]
Black phosphorus nanosheets	Small interfering RNA (siRNA) against PD-L1	MC38 and HCT116 colon cancer in mouse model	Induced chemo-phototherapeutic effects, including local reactive oxygen species and heat generation and necrosis/apoptosis, and interfered with PD-L1 pathway-regulated immune tolerance and inhibition of CD8^+^ T cells, prolonged the survival period.	[113]
Mesoporous silica NCs loaded with indoleamine 2,3-dioxygenase inhibitors	siRNA against PD-L1	Mouse model of prostate cancer	Exerted synergistic antitumor responses via immune checkpoint silencing and tumor microenvironment modulation	[128]

**Table 3:** A summary of currently ongoing clinical trials that are assessing the potential of various combinations of nanocarriers and immune checkpoint inhibitors.

Nanocarriers (NCs)	Immune Checkpoint Inhibitor(s)	Study Participants	Number of Participants	Phase of the Study	Clinical Trial ID
Nab-paclitaxel (a NC albumin-bound formulation of paclitaxel)	Durvalumab	Metastatic triple negative breast cancer (TNBC) patients	70	Phase II	NCT03606967
NBTXR3 (a radioenhancer composed of functionalized hafnium oxide NCs)	Nivolumab, pembrolizumab	Patients with advanced cancers	145	Phase I	NCT03589339
Nab-paclitaxel	Nivolumab	Early stage non-small cell lung cancer (NSCLC) patients	48	Phase II	NCT04015778
Nab-paclitaxel	Atezolizumab, bevacizumab	TNBC patients	91	Phase II	NCT03961698
Nab-paclitaxel	Pembrolizumab	HER-2 negative metastatic breast cancer	70	Phase II	NCT02752685
Nab-paclitaxel	HLX10	Patients with Stage IIIB/IIIC or IV NSCLC	537	Phase III	NCT04033354

## Data Availability

The original contributions presented in this study are included in the article. Further inquiries can be directed to the corresponding author.
